# A Milwaukee Test in Germany

**Published:** 1896-04

**Authors:** B. Fincke

**Affiliations:** Brooklyn, N. Y.


### A MILWAUKEE TEST IN GERMANY.

B. Fincke, M. D., Brooklyn, N. Y.

Dr. Schier, in Mainz, has for some time been proving a number of remedies, the last of which was Lolium-temulentum. (See *Allgem. Hom. Zeitung*, March 26th, 1896.) He gave it in doses of one to 350 drops of the mother tincture, and twice in a dose of five drops of the 1st dec. and five drops of the 2d dec. potencies.

After a synopsis of the symptoms obtained he continued:

"Though most of the provers have exerted themselves to gain a useful result with quite substantial doses, the product of the proving cannot well be called satisfactory. The reaction of the greatest part of the proving society was equal, and almost equal to zero in spite of the large doses. Those provers who experimented with potencies (two only using the 1st and 2d decs.) had been still less successful, and I believe the more we prove medicines, the more we come to the conviction that the symptoms of our materia medica, obtained with high potencies, are, for the most part, to be laid to pure imagination, called in modern parlance auto-suggestion. This, of course, is most unpleasant for the gentlemen who are advocates of high potencies, but I am ready to abide by the consequences of my assertion, and to furnish a strictly scientific proof according to everywhere acknowledged methods, to bear the cost of the test proposed by me, and besides to commit myself to pay a fine of 100 marks for a good homœopathic purpose if I am shown to be wrong. I, therefore, propose the following of the here-named twelve thoroughly proved polychrests: Aconite, Arsenicum, Belladonna, Bryonia, Calcarea-carb., China, Hepar-sulph-calc., Ipecacuanha, Mercurius-sol-hahn., Nux-vomica, Pulsatilla, Phosphorus. I shall send to every gentleman who announces himself willing to make the experiment, the to him unknown, but numerated remedy in the 30th–100th potency, according to my selection in the original packing ordered from the publisher of this journal with the request to prove it, to diagnosticate it by the symptoms obtained, and to send the diagnosis with description of symptoms under seal to one member of the commission, for which, by leave of the gentleman, I propose the Executive Committee of the Homœopathic Central-Verein. Simultaneously with the sending of the remedies, also under seal, provided with the corresponding number under the personal control of a colleague, I shall send the names of the respective remedies to the members of the Executive Committee of the Central-Verein. At the next meeting of the Central-Verein, the sealed-up diagnosis and names of the remedies are publicly compared, and I shall confess myself vanquished, if only ten results are presented and only five have hit the bull's-eye.*

"Should these experiments succeed, they would prove that 1. high potencies produce distinct and characteristic symptoms in the healthy; 2. that the correctly diagnosticated remedies are so thoroughly proved that the *experimentum crucis* succeeds—*i. e.*, that these remedies can be diagnosticated by their symptoms, a postulate which can be demanded by natural science. The proof of the assertion that high potencies in general act on the sick, or more energetically than low potencies would thereby, however, not be furnished; for in free nature we find throughout the confirmation of my view that in regard to the simillimum—*i. e.*, remedy—the sick organism is less susceptible to it—*i. e.*, it reacts less hostile to it than the healthy in regard to the same remedy.

"Here, therefore, is the imaginably best opportunity offered to those gentlemen who have an interest in the scientific progress of Homœopathy, and in my opinion, especially the gentlemen high potentialists should exhibit the sufficient ardor to prove the correctness of their views *coram publico*. *Hic Rhodus, hic salta!*

"Will the gentlemen dare to do it?

"If we consider that quite a great part of the remedies which we use is only proved with high potencies—Hahnemann and his believing disciples as is well known, never proved in the last time with potencies lower than 30th cent.—we should think that the interest for provings of remedies should be found stronger among the colleagues than this, alas! is the case. I find myself compelled to remark in this place once more expressly, that on my part none of the proving colleagues has been requested or importuned to prove only the mother-tincture; the participants of the proving receive only the mother-tincture from the pharmacy, and if they do not prefer to prepare the

---

* Since the action of a remedy, also in the mother-tincture, depends upon the *a priori* not-to-be-determined individual capacity of reaction of the prover in regard to this remedy, a possible want of action of the lower remedy in high potencies in a prover does not allow a conclusion upon the want of efficaciousness of these high potencies.—*Ed. Allg. Hom. Zeit.*

potencies which they desire themselves, they can also have the potencies from the pharmacy at their pleasure. I can indeed not imagine what else could be desired, and I do not see why I should disown my conviction of the better action of the strong doses which correspond to natural conditions; even by the results of the provings this has been decidedly corroborated. The gentlemen potentialists claim likewise the right of uttering their opinions freely in ample measure, and nothing is more desirable with me, than when the gentlemen take a very warm interest in the proposed test, and try to authenticate the correctness of their conceptions and by an argument consistent with the claims of natural science."

Thus far Dr. Schier. We have been there before, and we can wait with equanimity the outcome of this proposed test, because we have done the work of proving the efficacy of high potencies in health and disease according to scientific methods before. Nothing can convince a sceptic better than to make the trial himself, a sceptic is convinced that the strong doses of the mother-tincture act better than potencies derived from it on account of its correspondence to nature. His conviction can only be overturned by his own experiment, which then will have the more weight with him, because he can take all the precautions which science claims, even including the modern auto-suggestion to which he thinks high potentialists are peculiarly liable.

*Hic Rhodus, hic salta!*

*Ceterum censeo, macrodosiam esse delendam.*

